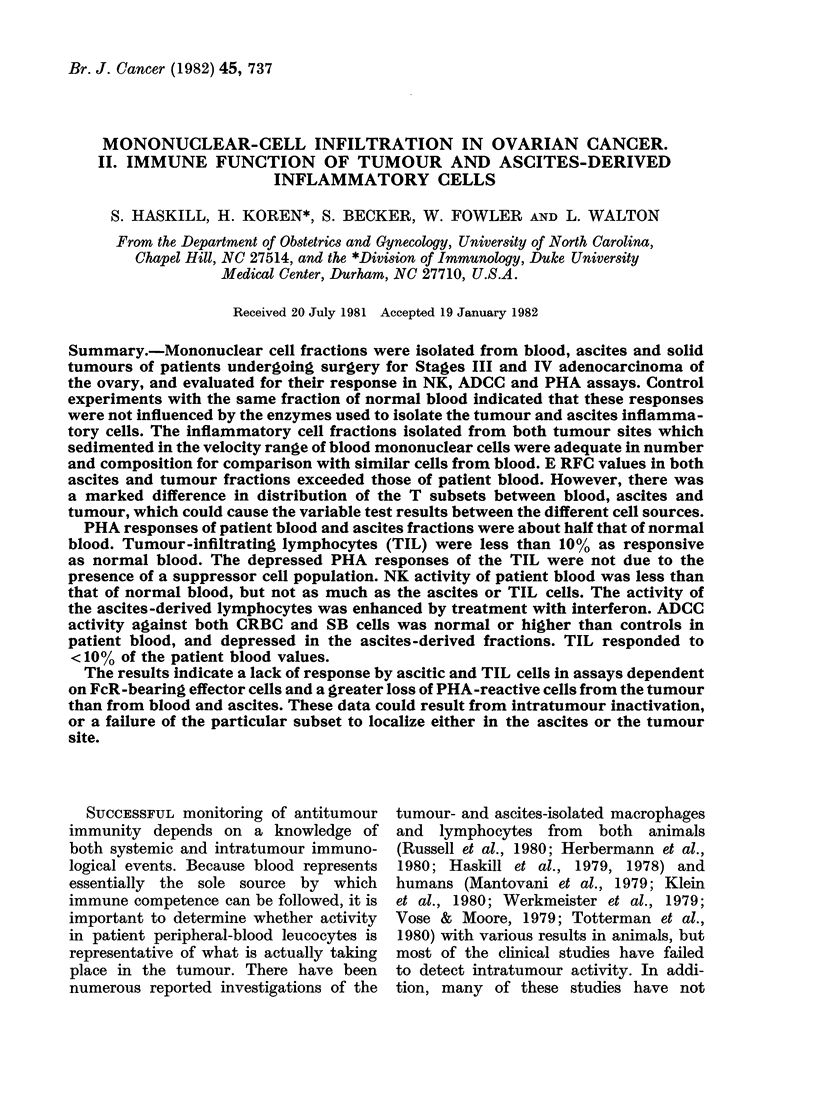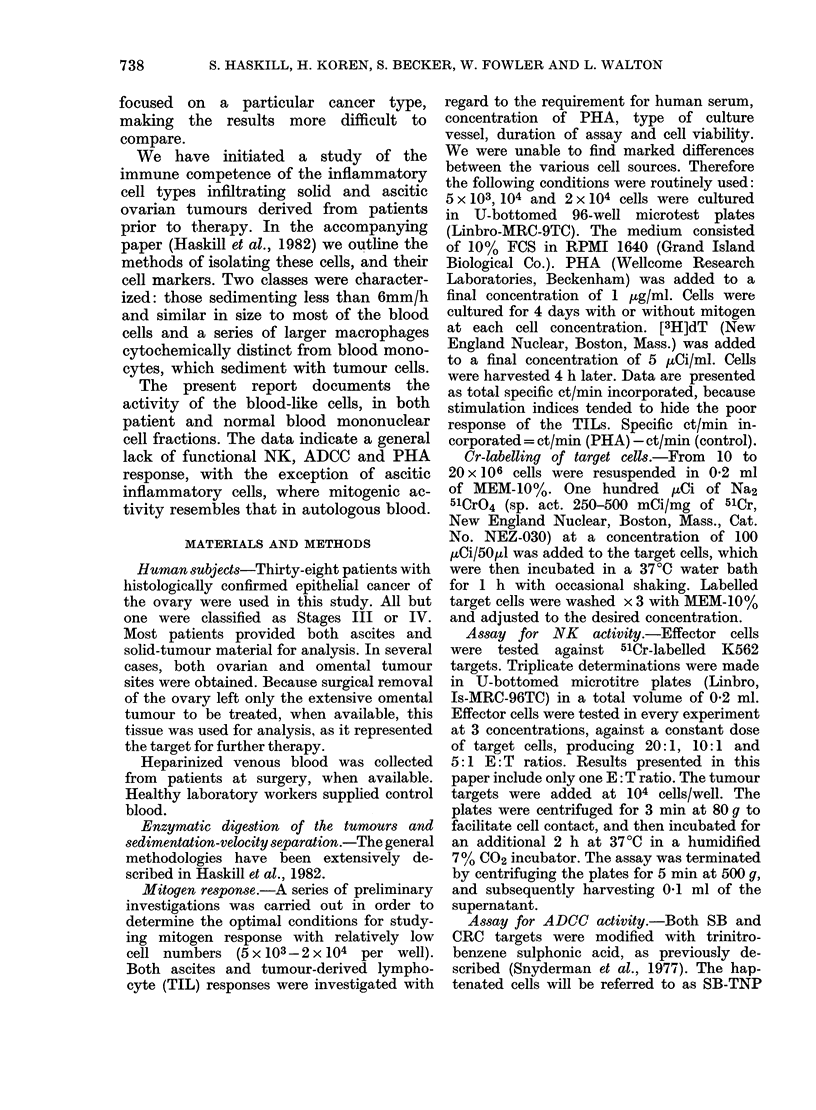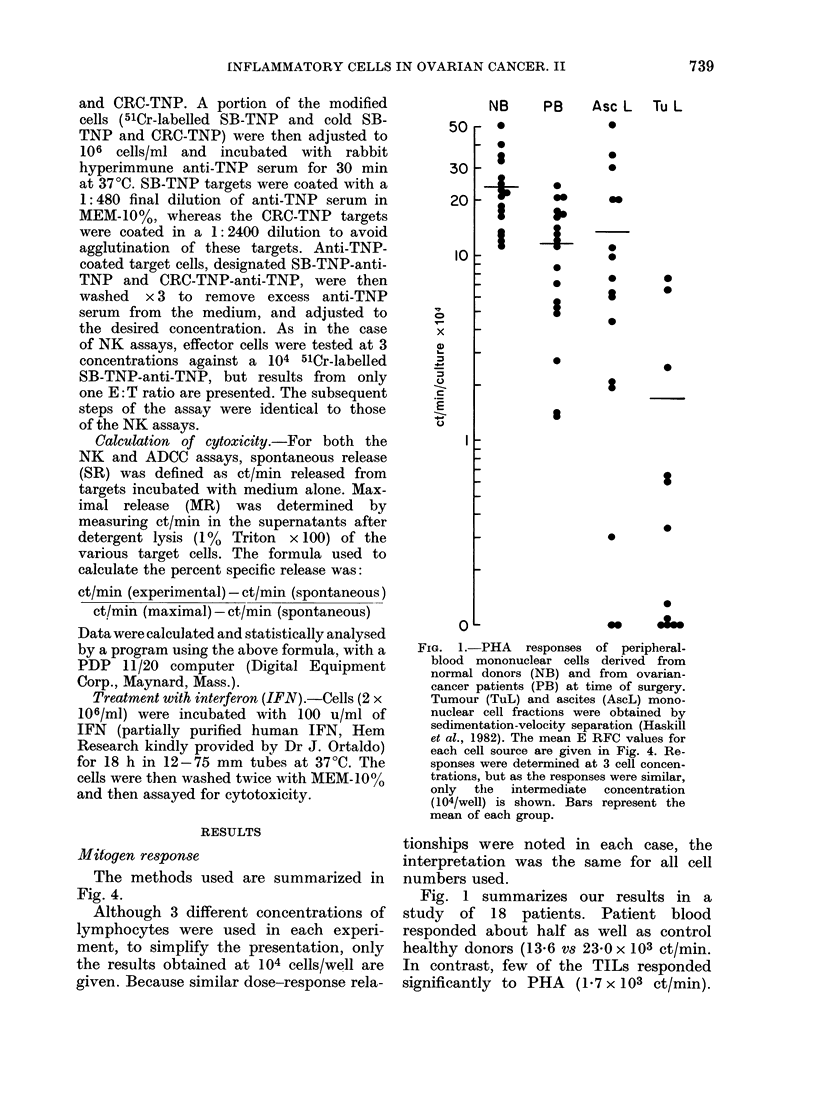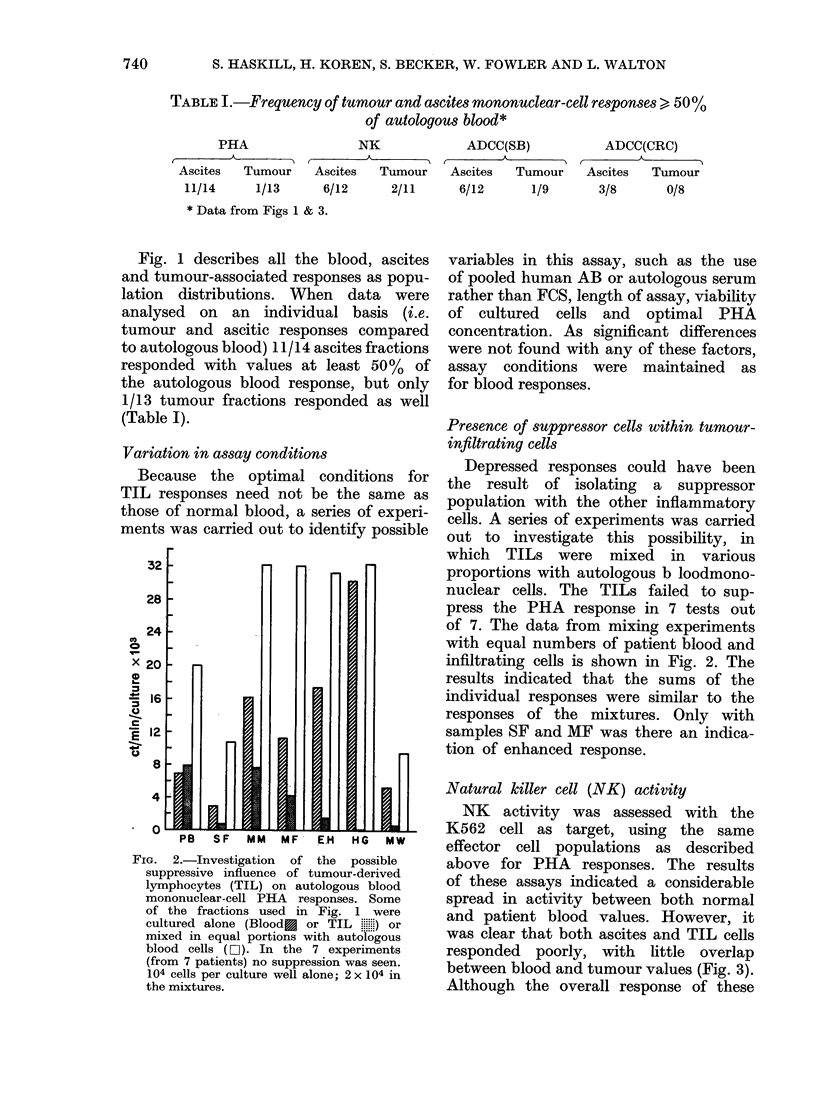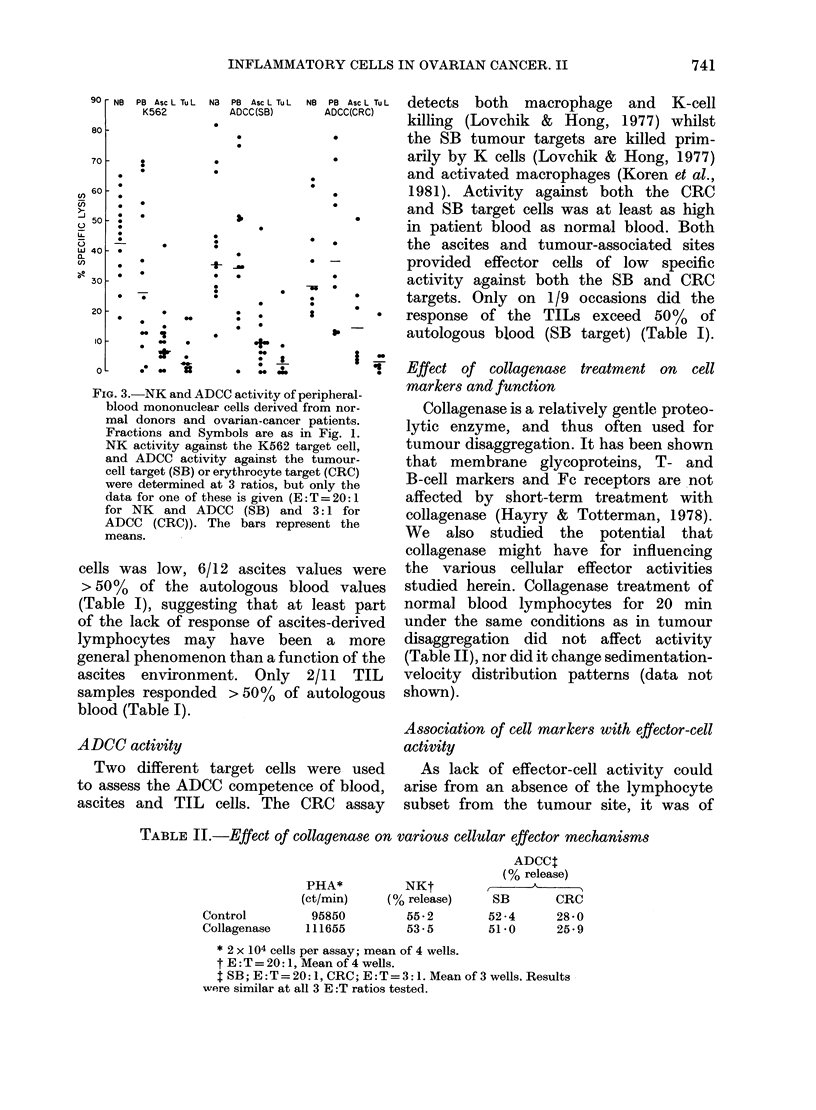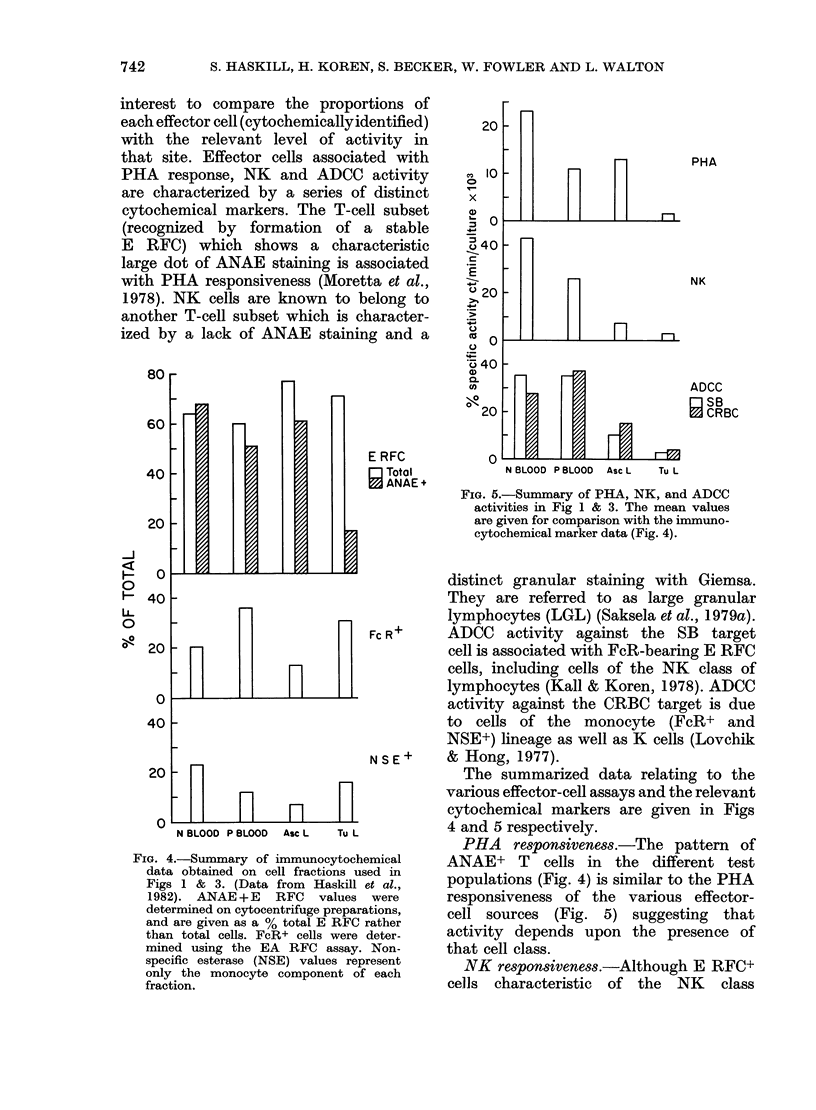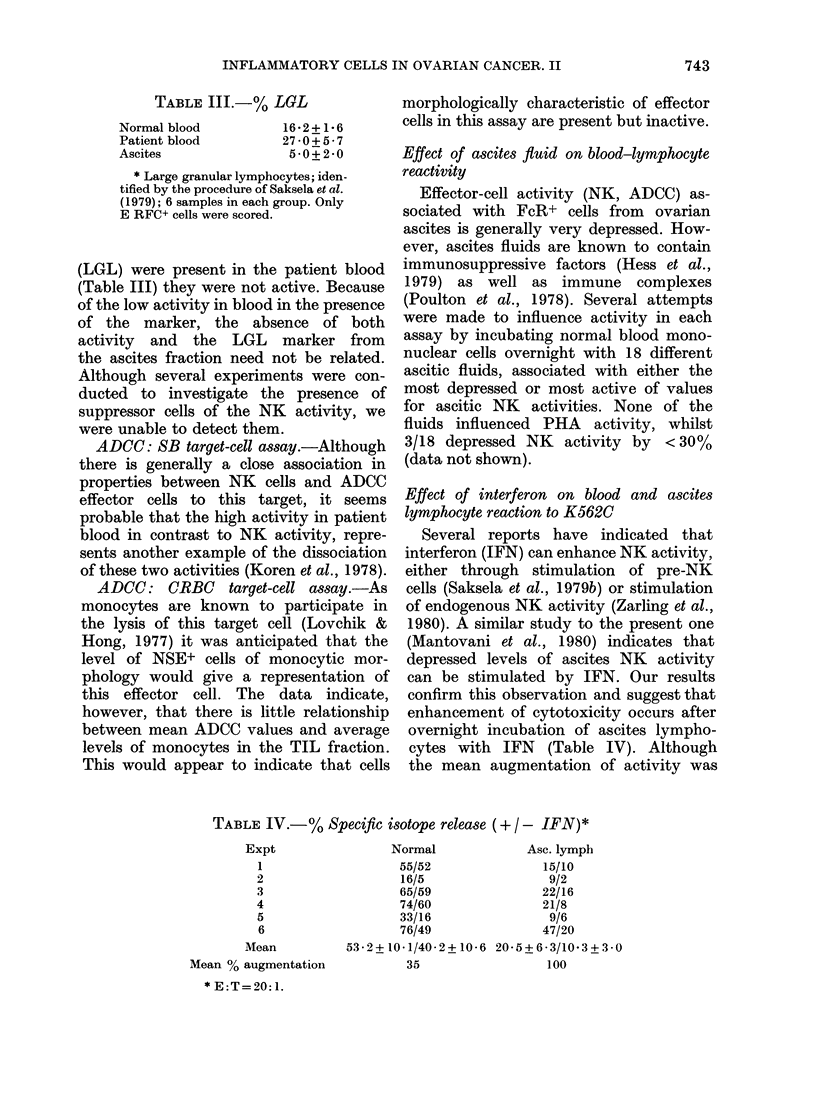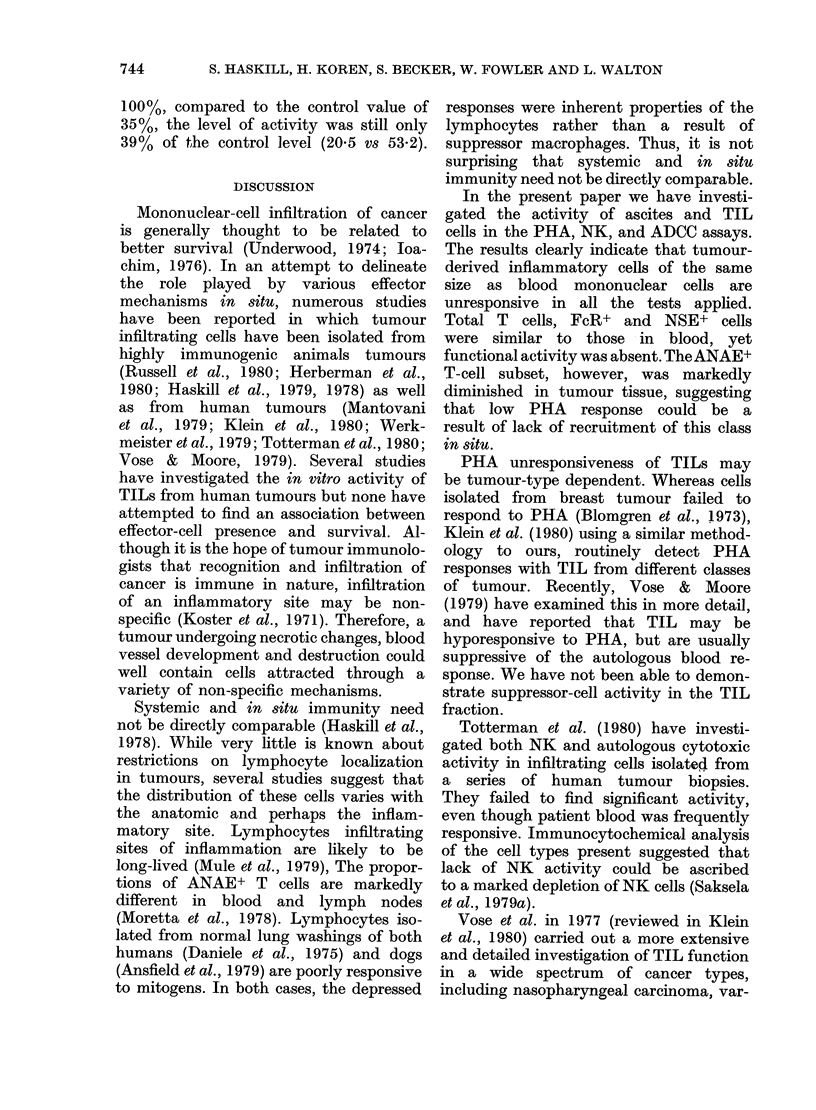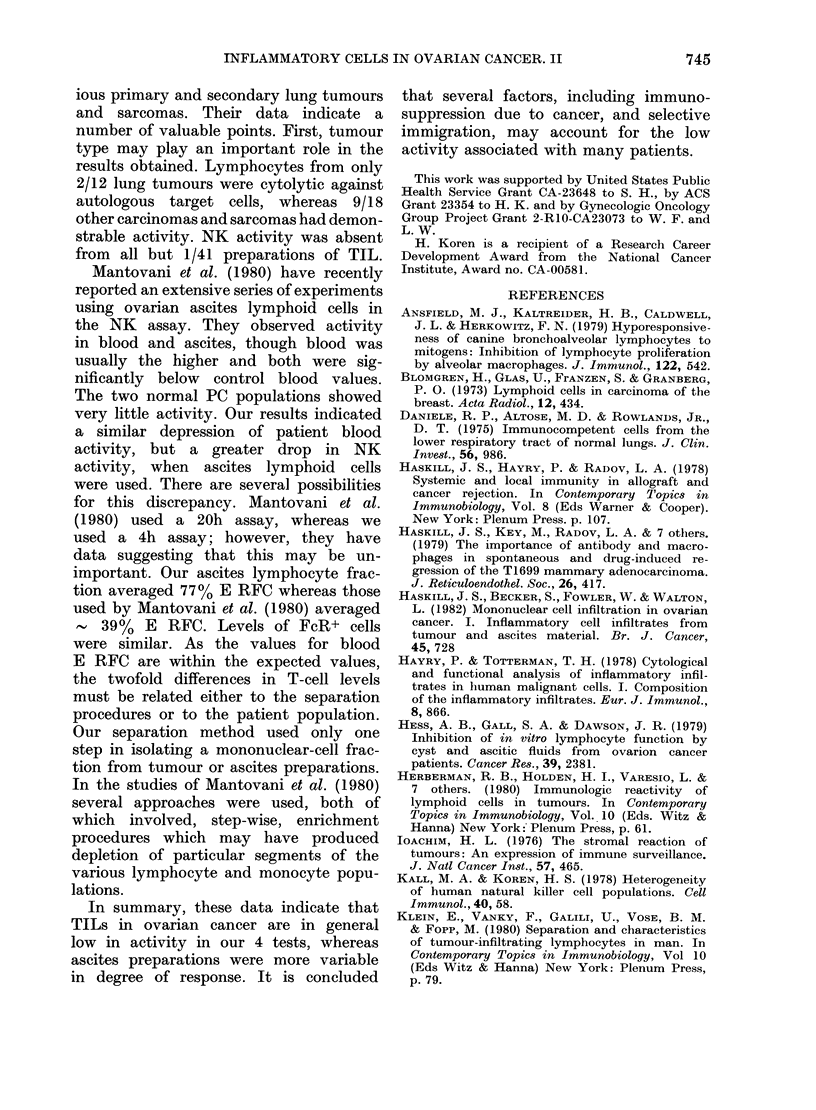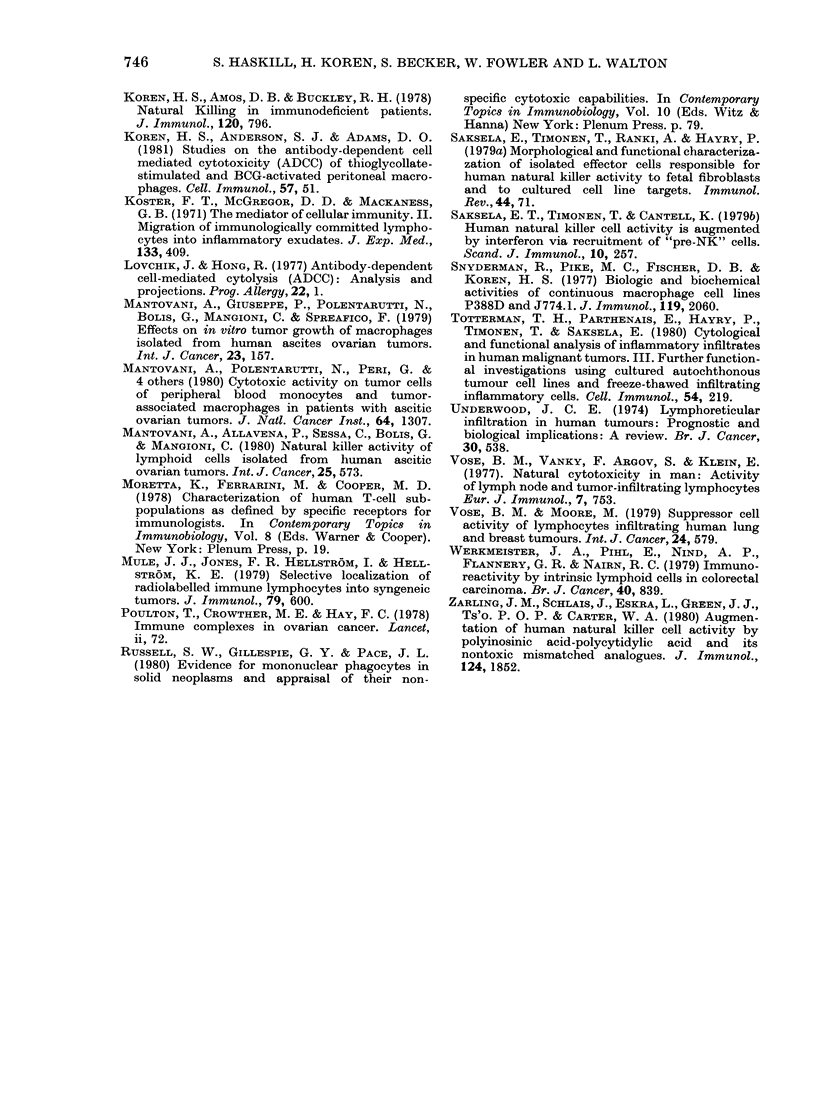# Mononuclear-cell infiltration in ovarian cancer. II. Immune function of tumour and ascites-derived inflammatory cells.

**DOI:** 10.1038/bjc.1982.115

**Published:** 1982-05

**Authors:** S. Haskill, H. Koren, S. Becker, W. Fowler, L. Walton

## Abstract

Mononuclear cell fractions were isolated from blood, ascites and solid tumours of patients undergoing surgery for Stages III and IV adenocarcinoma of the ovary, and evaluated for their response in NK, ADCC and PHA assays. Control experiments with the same fraction of normal blood indicated that these responses were not influenced by the enzymes used to isolate the tumour and ascites inflammatory cells. The inflammatory cell fractions isolated from both tumour sites which sedimented in the velocity range of blood mononuclear cells were adequate in number and composition for comparison with similar cells from blood. E RFC values in both ascites and tumour fractions exceeded those of patient blood. However, there was a marked difference in distribution of the T subsets between blood, ascites and tumour, which could cause the variable test results between the different cell sources. PHA responses of patient blood and ascites fractions were about half that of normal blood. Tumour-infiltrating lymphocytes (TIL) were less than 10% as responsive as normal blood. The depressed PHA responses of the TIL were not due to the presence of a suppressor cell population. NK activity of patient blood was less than that of normal blood, but not as much as the ascites of TIL cells. The activity of the ascites-derived lymphocytes was enhanced by treatment with interferon. ADCC activity against both CRBC and SB cells was normal or higher than controls in patient blood, and depressed in the ascites-derived fractions. TIL responded to less than 10% of the patient blood values. The results indicate a lack of response by ascitic and TIL cells in assays dependent on FcR-bearing effector cells and a greater loss of PHA-reactive cells from the tumour than from blood and ascites. These data could result from intratumour inactivation, or a failure of the particular subset to localize either in the ascites or the tumour site.


*Br. J. Cancer* (1982) **45**, 737

### MONONUCLEAR-CELL INFILTRATION IN OVARIAN CANCER. II. IMMUNE FUNCTION OF TUMOUR AND ASCITES-DERIVED INFLAMMATORY CELLS

S. HASKILL, H. KOREN*, S. BECKER, W. FOWLER AND L. WALTON

*From the Department of Obstetrics and Gynecology, University of North Carolina, Chapel Hill, NC* 27514, *and the *Division of Immunology, Duke University Medical Center, Durham, NC* 27710, *U.S.A.*

Received 20 July 1981 Accepted 19 January 1982

**Summary.—Mononuclear cell fractions were isolated from blood, ascites and solid tumours of patients undergoing surgery for Stages III and IV adenocarcinoma of the ovary, and evaluated for their response in NK, ADCC and PHA assays. Control experiments with the same fraction of normal blood indicated that these responses were not influenced by the enzymes used to isolate the tumour and ascites inflammatory cells. The inflammatory cell fractions isolated from both tumour sites which sedimented in the velocity range of blood mononuclear cells were adequate in number and composition for comparison with similar cells from blood. E RFC values in both ascites and tumour fractions exceeded those of patient blood. However, there was a marked difference in distribution of the T subsets between blood, ascites and tumour, which could cause the variable test results between the different cell sources.**

**PHA responses of patient blood and ascites fractions were about half that of normal blood. Tumour-infiltrating lymphocytes (TIL) were less than 10% as responsive as normal blood. The depressed PHA responses of the TIL were not due to the presence of a suppressor cell population. NK activity of patient blood was less than that of normal blood, but not as much as the ascites or TIL cells. The activity of the ascites-derived lymphocytes was enhanced by treatment with interferon. ADCC activity against both CRBC and SB cells was normal or higher than controls in patient blood, and depressed in the ascites-derived fractions. TIL responded to <10% of the patient blood values.**

**The results indicate a lack of response by ascitic and TIL cells in assays dependent on FcR-bearing effector cells and a greater loss of PHA-reactive cells from the tumour than from blood and ascites. These data could result from intratumour inactivation, or a failure of the particular subset to localize either in the ascites or the tumour site.**

SUCCESSFUL monitoring of antitumour immunity depends on a knowledge of both systemic and intratumour immunological events. Because blood represents essentially the sole source by which immune competence can be followed, it is important to determine whether activity in patient peripheral-blood leucocytes is representative of what is actually taking place in the tumour. There have been numerous reported investigations of the tumour- and ascites-isolated macrophages and lymphocytes from both animals (Russell *et al.*, 1980; Herbermann *et al.*, 1980; Haskill *et al.*, 1979, 1978) and humans (Mantovani *et al.*, 1979; Klein *et al.*, 1980; Werkmeister *et al.*, 1979; Vose & Moore, 1979; Totterman *et al.*, 1980) with various results in animals, but most of the clinical studies have failed to detect intratumour activity. In addition, many of these studies have not focused on a particular cancer type, making the results more difficult to compare.

We have initiated a study of the immune competence of the inflammatory cell types infiltrating solid and ascitic ovarian tumours derived from patients prior to therapy. In the accompanying paper (Haskill *et al.*, 1982) we outline the methods of isolating these cells, and their cell markers. Two classes were characterized: those sedimenting less than 6mm/h and similar in size to most of the blood cells and a series of larger macrophages cytochemically distinct from blood monocytes, which sediment with tumour cells.

The present report documents the activity of the blood-like cells, in both patient and normal blood mononuclear cell fractions. The data indicate a general lack of functional NK, ADCC and PHA response, with the exception of ascitic inflammatory cells, where mitogenic activity resembles that in autologous blood.

### MATERIALS AND METHODS

*Human subjects*—Thirty-eight patients with histologically confirmed epithelial cancer of the ovary were used in this study. All but one were classified as Stages III or IV. Most patients provided both ascites and solid-tumour material for analysis. In several cases, both ovarian and omental tumour sites were obtained. Because surgical removal of the ovary left only the extensive omental tumour to be treated, when available, this tissue was used for analysis, as it represented the target for further therapy.

Heparinized venous blood was collected from patients at surgery, when available. Healthy laboratory workers supplied control blood.

*Enzymatic digestion of the tumours and sedimentation-velocity separation.*—The general methodologies have been extensively described in Haskill *et al.*, 1982.

*Mitogen response.*—A series of preliminary investigations was carried out in order to determine the optimal conditions for studying mitogen response with relatively low cell numbers ($5\times10^3-2\times10^4$ per well). Both ascites and tumour-derived lymphocyte (TIL) responses were investigated with regard to the requirement for human serum, concentration of PHA, type of culture vessel, duration of assay and cell viability. We were unable to find marked differences between the various cell sources. Therefore the following conditions were routinely used: $5\times10^3$, $10^4$ and $2\times10^4$ cells were cultured in U-bottomed 96-well microtest plates (Linbro-MRC-9TC). The medium consisted of 10% FCS in RPMI 1640 (Grand Island Biological Co.). PHA (Wellcome Research Laboratories, Beckenham) was added to a final concentration of 1 $\mu$g/ml. Cells were cultured for 4 days with or without mitogen at each cell concentration. [$^3$H]dT (New England Nuclear, Boston, Mass.) was added to a final concentration of 5 $\mu$Ci/ml. Cells were harvested 4 h later. Data are presented as total specific ct/min incorporated, because stimulation indices tended to hide the poor response of the TILs. Specific ct/min incorporated = ct/min (PHA) − ct/min (control).

*Cr-labelling of target cells.*—From 10 to $20\times10^6$ cells were resuspended in 0·2 ml of MEM-10%. One hundred $\mu$Ci of $Na_2$ $^{51}CrO_4$ (sp. act. 250–500 mCi/mg of $^{51}$Cr, New England Nuclear, Boston, Mass., Cat. No. NEZ-030) at a concentration of 100 $\mu$Ci/50$\mu$l was added to the target cells, which were then incubated in a 37°C water bath for 1 h with occasional shaking. Labelled target cells were washed ×3 with MEM-10% and adjusted to the desired concentration.

*Assay for NK activity.*—Effector cells were tested against $^{51}$Cr-labelled K562 targets. Triplicate determinations were made in U-bottomed microtitre plates (Linbro, Is-MRC-96TC) in a total volume of 0·2 ml. Effector cells were tested in every experiment at 3 concentrations, against a constant dose of target cells, producing 20:1, 10:1 and 5:1 E:T ratios. Results presented in this paper include only one E:T ratio. The tumour targets were added at $10^4$ cells/well. The plates were centrifuged for 3 min at 80 *g* to facilitate cell contact, and then incubated for an additional 2 h at 37°C in a humidified 7% $CO_2$ incubator. The assay was terminated by centrifuging the plates for 5 min at 500 *g*, and subsequently harvesting 0·1 ml of the supernatant.

*Assay for ADCC activity.*—Both SB and CRC targets were modified with trinitrobenzene sulphonic acid, as previously described (Snyderman *et al.*, 1977). The haptenated cells will be referred to as SB-TNP

and CRC-TNP. A portion of the modified cells ($^{51}$Cr-labelled SB-TNP and cold SB-TNP and CRC-TNP) were then adjusted to $10^6$ cells/ml and incubated with rabbit hyperimmune anti-TNP serum for 30 min at 37 °C. SB-TNP targets were coated with a 1:480 final dilution of anti-TNP serum in MEM-10%, whereas the CRC-TNP targets were coated in a 1:2400 dilution to avoid agglutination of these targets. Anti-TNP-coated target cells, designated SB-TNP-anti-TNP and CRC-TNP-anti-TNP, were then washed ×3 to remove excess anti-TNP serum from the medium, and adjusted to the desired concentration. As in the case of NK assays, effector cells were tested at 3 concentrations against a $10^4$ $^{51}$Cr-labelled SB-TNP-anti-TNP, but results from only one E:T ratio are presented. The subsequent steps of the assay were identical to those of the NK assays.

*Calculation of cytoxicity.*—For both the NK and ADCC assays, spontaneous release (SR) was defined as ct/min released from targets incubated with medium alone. Maximal release (MR) was determined by measuring ct/min in the supernatants after detergent lysis (1% Triton ×100) of the various target cells. The formula used to calculate the percent specific release was:

$$\frac{\text{ct/min (experimental)} - \text{ct/min (spontaneous)}}{\text{ct/min (maximal)} - \text{ct/min (spontaneous)}}$$

Data were calculated and statistically analysed by a program using the above formula, with a PDP 11/20 computer (Digital Equipment Corp., Maynard, Mass.).

*Treatment with interferon (IFN).*—Cells (2 × $10^6$/ml) were incubated with 100 u/ml of IFN (partially purified human IFN, Hem Research kindly provided by Dr J. Ortaldo) for 18 h in 12–75 mm tubes at 37 °C. The cells were then washed twice with MEM-10% and then assayed for cytotoxicity.

FIG. 1.—PHA responses of peripheral-blood mononuclear cells derived from normal donors (NB) and from ovarian-cancer patients (PB) at time of surgery. Tumour (TuL) and ascites (AscL) mononuclear cell fractions were obtained by sedimentation-velocity separation (Haskill *et al.*, 1982). The mean E RFC values for each cell source are given in Fig. 4. Responses were determined at 3 cell concentrations, but as the responses were similar, only the intermediate concentration ($10^4$/well) is shown. Bars represent the mean of each group.

### RESULTS

### *Mitogen response*

The methods used are summarized in Fig. 4.

Although 3 different concentrations of lymphocytes were used in each experiment, to simplify the presentation, only the results obtained at $10^4$ cells/well are given. Because similar dose–response relationships were noted in each case, the interpretation was the same for all cell numbers used.

Fig. 1 summarizes our results in a study of 18 patients. Patient blood responded about half as well as control healthy donors (13·6 *vs* 23·0 × $10^3$ ct/min. In contrast, few of the TILs responded significantly to PHA (1·7 × $10^3$ ct/min).

TABLE I.—*Frequency of tumour and ascites mononuclear-cell responses ≥ 50% of autologous blood**

| PHA | | NK | | ADCC(SB) | | ADCC(CRC) | |
|---|---|---|---|---|---|---|---|
| Ascites | Tumour | Ascites | Tumour | Ascites | Tumour | Ascites | Tumour |
| 11/14 | 1/13 | 6/12 | 2/11 | 6/12 | 1/9 | 3/8 | 0/8 |

* Data from Figs 1 & 3.

Fig. 1 describes all the blood, ascites and tumour-associated responses as population distributions. When data were analysed on an individual basis (*i.e.* tumour and ascitic responses compared to autologous blood) 11/14 ascites fractions responded with values at least 50% of the autologous blood response, but only 1/13 tumour fractions responded as well (Table I).

*Variation in assay conditions*

Because the optimal conditions for TIL responses need not be the same as those of normal blood, a series of experiments was carried out to identify possible variables in this assay, such as the use of pooled human AB or autologous serum rather than FCS, length of assay, viability of cultured cells and optimal PHA concentration. As significant differences were not found with any of these factors, assay conditions were maintained as for blood responses.

FIG. 2.—Investigation of the possible suppressive influence of tumour-derived lymphocytes (TIL) on autologous blood mononuclear-cell PHA responses. Some of the fractions used in Fig. 1 were cultured alone (Blood ▨ or TIL ▦) or mixed in equal portions with autologous blood cells (□). In the 7 experiments (from 7 patients) no suppression was seen. $10^4$ cells per culture well alone; $2 \times 10^4$ in the mixtures.

*Presence of suppressor cells within tumour-infiltrating cells*

Depressed responses could have been the result of isolating a suppressor population with the other inflammatory cells. A series of experiments was carried out to investigate this possibility, in which TILs were mixed in various proportions with autologous b loodmononuclear cells. The TILs failed to suppress the PHA response in 7 tests out of 7. The data from mixing experiments with equal numbers of patient blood and infiltrating cells is shown in Fig. 2. The results indicated that the sums of the individual responses were similar to the responses of the mixtures. Only with samples SF and MF was there an indication of enhanced response.

*Natural killer cell (NK) activity*

NK activity was assessed with the K562 cell as target, using the same effector cell populations as described above for PHA responses. The results of these assays indicated a considerable spread in activity between both normal and patient blood values. However, it was clear that both ascites and TIL cells responded poorly, with little overlap between blood and tumour values (Fig. 3). Although the overall response of these

FIG. 3.—NK and ADCC activity of peripheral-blood mononuclear cells derived from normal donors and ovarian-cancer patients. Fractions and Symbols are as in Fig. 1. NK activity against the K562 target cell, and ADCC activity against the tumour-cell target (SB) or erythrocyte target (CRC) were determined at 3 ratios, but only the data for one of these is given (E:T = 20:1 for NK and ADCC (SB) and 3:1 for ADCC (CRC)). The bars represent the means.

cells was low, 6/12 ascites values were > 50% of the autologous blood values (Table I), suggesting that at least part of the lack of response of ascites-derived lymphocytes may have been a more general phenomenon than a function of the ascites environment. Only 2/11 TIL samples responded > 50% of autologous blood (Table I).

### *ADCC activity*

Two different target cells were used to assess the ADCC competence of blood, ascites and TIL cells. The CRC assay detects both macrophage and K-cell killing (Lovchik & Hong, 1977) whilst the SB tumour targets are killed primarily by K cells (Lovchik & Hong, 1977) and activated macrophages (Koren *et al.*, 1981). Activity against both the CRC and SB target cells was at least as high in patient blood as normal blood. Both the ascites and tumour-associated sites provided effector cells of low specific activity against both the SB and CRC targets. Only on 1/9 occasions did the response of the TILs exceed 50% of autologous blood (SB target) (Table I).

### *Effect of collagenase treatment on cell markers and function*

Collagenase is a relatively gentle proteolytic enzyme, and thus often used for tumour disaggregation. It has been shown that membrane glycoproteins, T- and B-cell markers and Fc receptors are not affected by short-term treatment with collagenase (Hayry & Totterman, 1978). We also studied the potential that collagenase might have for influencing the various cellular effector activities studied herein. Collagenase treatment of normal blood lymphocytes for 20 min under the same conditions as in tumour disaggregation did not affect activity (Table II), nor did it change sedimentation-velocity distribution patterns (data not shown).

### *Association of cell markers with effector-cell activity*

As lack of effector-cell activity could arise from an absence of the lymphocyte subset from the tumour site, it was of

TABLE II.—*Effect of collagenase on various cellular effector mechanisms*

| | PHA* (ct/min) | NK† (% release) | ADCC‡ (% release) SB | CRC |
|---|---|---|---|---|
| Control | 95850 | 55·2 | 52·4 | 28·0 |
| Collagenase | 111655 | 53·5 | 51·0 | 25·9 |

* $2 \times 10^4$ cells per assay; mean of 4 wells.
† E:T = 20:1, Mean of 4 wells.
‡ SB; E:T = 20:1, CRC; E:T = 3:1. Mean of 3 wells. Results were similar at all 3 E:T ratios tested.

interest to compare the proportions of each effector cell (cytochemically identified) with the relevant level of activity in that site. Effector cells associated with PHA response, NK and ADCC activity are characterized by a series of distinct cytochemical markers. The T-cell subset (recognized by formation of a stable E RFC) which shows a characteristic large dot of ANAE staining is associated with PHA responsiveness (Moretta *et al.*, 1978). NK cells are known to belong to another T-cell subset which is characterized by a lack of ANAE staining and a

FIG. 4.—Summary of immunocytochemical data obtained on cell fractions used in Figs 1 & 3. (Data from Haskill *et al.*, 1982). ANAE+E RFC values were determined on cytocentrifuge preparations, and are given as a % total E RFC rather than total cells. FcR+ cells were determined using the EA RFC assay. Non-specific esterase (NSE) values represent only the monocyte component of each fraction.

FIG. 5.—Summary of PHA, NK, and ADCC activities in Fig 1 & 3. The mean values are given for comparison with the immunocytochemical marker data (Fig. 4).

distinct granular staining with Giemsa. They are referred to as large granular lymphocytes (LGL) (Saksela *et al.*, 1979*a*). ADCC activity against the SB target cell is associated with FcR-bearing E RFC cells, including cells of the NK class of lymphocytes (Kall & Koren, 1978). ADCC activity against the CRBC target is due to cells of the monocyte ($FcR^+$ and $NSE^+$) lineage as well as K cells (Lovchik & Hong, 1977).

The summarized data relating to the various effector-cell assays and the relevant cytochemical markers are given in Figs 4 and 5 respectively.

*PHA responsiveness.*—The pattern of $ANAE^+$ T cells in the different test populations (Fig. 4) is similar to the PHA responsiveness of the various effector-cell sources (Fig. 5) suggesting that activity depends upon the presence of that cell class.

*NK responsiveness.*—Although E $RFC^+$ cells characteristic of the NK class

TABLE III.—% *LGL*

| | |
|---|---|
| Normal blood | $16 \cdot 2 \pm 1 \cdot 6$ |
| Patient blood | $27 \cdot 0 \pm 5 \cdot 7$ |
| Ascites | $5 \cdot 0 \pm 2 \cdot 0$ |

* Large granular lymphocytes; identified by the procedure of Saksela *et al.* (1979); 6 samples in each group. Only E RFC+ cells were scored.

(LGL) were present in the patient blood (Table III) they were not active. Because of the low activity in blood in the presence of the marker, the absence of both activity and the LGL marker from the ascites fraction need not be related. Although several experiments were conducted to investigate the presence of suppressor cells of the NK activity, we were unable to detect them.

*ADCC: SB target-cell assay.*—Although there is generally a close association in properties between NK cells and ADCC effector cells to this target, it seems probable that the high activity in patient blood in contrast to NK activity, represents another example of the dissociation of these two activities (Koren *et al.*, 1978).

*ADCC: CRBC target-cell assay.*—As monocytes are known to participate in the lysis of this target cell (Lovchik & Hong, 1977) it was anticipated that the level of NSE+ cells of monocytic morphology would give a representation of this effector cell. The data indicate, however, that there is little relationship between mean ADCC values and average levels of monocytes in the TIL fraction. This would appear to indicate that cells morphologically characteristic of effector cells in this assay are present but inactive.

*Effect of ascites fluid on blood–lymphocyte reactivity*

Effector-cell activity (NK, ADCC) associated with FcR+ cells from ovarian ascites is generally very depressed. However, ascites fluids are known to contain immunosuppressive factors (Hess *et al.*, 1979) as well as immune complexes (Poulton *et al.*, 1978). Several attempts were made to influence activity in each assay by incubating normal blood mononuclear cells overnight with 18 different ascitic fluids, associated with either the most depressed or most active of values for ascitic NK activities. None of the fluids influenced PHA activity, whilst 3/18 depressed NK activity by $<30\%$ (data not shown).

*Effect of interferon on blood and ascites lymphocyte reaction to K562C*

Several reports have indicated that interferon (IFN) can enhance NK activity, either through stimulation of pre-NK cells (Saksela *et al.*, 1979*b*) or stimulation of endogenous NK activity (Zarling *et al.*, 1980). A similar study to the present one (Mantovani *et al.*, 1980) indicates that depressed levels of ascites NK activity can be stimulated by IFN. Our results confirm this observation and suggest that enhancement of cytotoxicity occurs after overnight incubation of ascites lymphocytes with IFN (Table IV). Although the mean augmentation of activity was

TABLE IV.—% *Specific isotope release* (+/− *IFN*)*

| Expt | Normal | Asc. lymph |
|---|---|---|
| 1 | 55/52 | 15/10 |
| 2 | 16/5 | 9/2 |
| 3 | 65/59 | 22/16 |
| 4 | 74/60 | 21/8 |
| 5 | 33/16 | 9/6 |
| 6 | 76/49 | 47/20 |
| Mean | $53 \cdot 2 \pm 10 \cdot 1/40 \cdot 2 \pm 10 \cdot 6$ | $20 \cdot 5 \pm 6 \cdot 3/10 \cdot 3 \pm 3 \cdot 0$ |
| Mean % augmentation | 35 | 100 |

* E:T = 20:1.

100%, compared to the control value of 35%, the level of activity was still only 39% of the control level (20·5 *vs* 53·2).

### DISCUSSION

Mononuclear-cell infiltration of cancer is generally thought to be related to better survival (Underwood, 1974; Ioachim, 1976). In an attempt to delineate the role played by various effector mechanisms *in situ*, numerous studies have been reported in which tumour infiltrating cells have been isolated from highly immunogenic animals tumours (Russell *et al.*, 1980; Herberman *et al.*, 1980; Haskill *et al.*, 1979, 1978) as well as from human tumours (Mantovani *et al.*, 1979; Klein *et al.*, 1980; Werkmeister *et al.*, 1979; Totterman *et al.*, 1980; Vose & Moore, 1979). Several studies have investigated the *in vitro* activity of TILs from human tumours but none have attempted to find an association between effector-cell presence and survival. Although it is the hope of tumour immunologists that recognition and infiltration of cancer is immune in nature, infiltration of an inflammatory site may be non-specific (Koster *et al.*, 1971). Therefore, a tumour undergoing necrotic changes, blood vessel development and destruction could well contain cells attracted through a variety of non-specific mechanisms.

Systemic and *in situ* immunity need not be directly comparable (Haskill *et al.*, 1978). While very little is known about restrictions on lymphocyte localization in tumours, several studies suggest that the distribution of these cells varies with the anatomic and perhaps the inflammatory site. Lymphocytes infiltrating sites of inflammation are likely to be long-lived (Mule *et al.*, 1979), The proportions of ANAE$^+$ T cells are markedly different in blood and lymph nodes (Moretta *et al.*, 1978). Lymphocytes isolated from normal lung washings of both humans (Daniele *et al.*, 1975) and dogs (Ansfield *et al.*, 1979) are poorly responsive to mitogens. In both cases, the depressed responses were inherent properties of the lymphocytes rather than a result of suppressor macrophages. Thus, it is not surprising that systemic and *in situ* immunity need not be directly comparable.

In the present paper we have investigated the activity of ascites and TIL cells in the PHA, NK, and ADCC assays. The results clearly indicate that tumour-derived inflammatory cells of the same size as blood mononuclear cells are unresponsive in all the tests applied. Total T cells, FcR$^+$ and NSE$^+$ cells were similar to those in blood, yet functional activity was absent. The ANAE$^+$ T-cell subset, however, was markedly diminished in tumour tissue, suggesting that low PHA response could be a result of lack of recruitment of this class *in situ*.

PHA unresponsiveness of TILs may be tumour-type dependent. Whereas cells isolated from breast tumour failed to respond to PHA (Blomgren *et al.*, 1973), Klein *et al.* (1980) using a similar methodology to ours, routinely detect PHA responses with TIL from different classes of tumour. Recently, Vose & Moore (1979) have examined this in more detail, and have reported that TIL may be hyporesponsive to PHA, but are usually suppressive of the autologous blood response. We have not been able to demonstrate suppressor-cell activity in the TIL fraction.

Totterman *et al.* (1980) have investigated both NK and autologous cytotoxic activity in infiltrating cells isolated from a series of human tumour biopsies. They failed to find significant activity, even though patient blood was frequently responsive. Immunocytochemical analysis of the cell types present suggested that lack of NK activity could be ascribed to a marked depletion of NK cells (Saksela *et al.*, 1979*a*).

Vose *et al.* in 1977 (reviewed in Klein *et al.*, 1980) carried out a more extensive and detailed investigation of TIL function in a wide spectrum of cancer types, including nasopharyngeal carcinoma, various primary and secondary lung tumours and sarcomas. Their data indicate a number of valuable points. First, tumour type may play an important role in the results obtained. Lymphocytes from only 2/12 lung tumours were cytolytic against autologous target cells, whereas 9/18 other carcinomas and sarcomas had demonstrable activity. NK activity was absent from all but 1/41 preparations of TIL.

Mantovani *et al.* (1980) have recently reported an extensive series of experiments using ovarian ascites lymphoid cells in the NK assay. They observed activity in blood and ascites, though blood was usually the higher and both were significantly below control blood values. The two normal PC populations showed very little activity. Our results indicated a similar depression of patient blood activity, but a greater drop in NK activity, when ascites lymphoid cells were used. There are several possibilities for this discrepancy. Mantovani *et al.* (1980) used a 20h assay, whereas we used a 4h assay; however, they have data suggesting that this may be unimportant. Our ascites lymphocyte fraction averaged 77% E RFC whereas those used by Mantovani *et al.* (1980) averaged ~ 39% E RFC. Levels of $FcR^+$ cells were similar. As the values for blood E RFC are within the expected values, the twofold differences in T-cell levels must be related either to the separation procedures or to the patient population. Our separation method used only one step in isolating a mononuclear-cell fraction from tumour or ascites preparations. In the studies of Mantovani *et al.* (1980) several approaches were used, both of which involved, step-wise, enrichment procedures which may have produced depletion of particular segments of the various lymphocyte and monocyte populations.

In summary, these data indicate that TILs in ovarian cancer are in general low in activity in our 4 tests, whereas ascites preparations were more variable in degree of response. It is concluded that several factors, including immunosuppression due to cancer, and selective immigration, may account for the low activity associated with many patients.

This work was supported by United States Public Health Service Grant CA-23648 to S. H., by ACS Grant 23354 to H. K. and by Gynecologic Oncology Group Project Grant 2-R10-CA23073 to W. F. and L. W.

H. Koren is a recipient of a Research Career Development Award from the National Cancer Institute, Award no. CA-00581.

### REFERENCES

ANSFIELD, M. J., KALTREIDER, H. B., CALDWELL, J. L. & HERKOWITZ, F. N. (1979) Hyporesponsiveness of canine bronchoalveolar lymphocytes to mitogens: Inhibition of lymphocyte proliferation by alveolar macrophages. *J. Immunol.*, **122**, 542.

BLOMGREN, H., GLAS, U., FRANZEN, S. & GRANBERG, P. O. (1973) Lymphoid cells in carcinoma of the breast. *Acta Radiol.*, **12**, 434.

DANIELE, R. P., ALTOSE, M. D. & ROWLANDS, JR., D. T. (1975) Immunocompetent cells from the lower respiratory tract of normal lungs. *J. Clin. Invest.*, **56**, 986.

HASKILL, J. S., HAYRY, P. & RADOV, L. A. (1978) Systemic and local immunity in allograft and cancer rejection. In *Contemporary Topics in Immunobiology*, Vol. 8 (Eds Warner & Cooper). New York: Plenum Press. p. 107.

HASKILL, J. S., KEY, M., RADOV, L. A. & 7 others. (1979) The importance of antibody and macrophages in spontaneous and drug-induced regression of the T1699 mammary adenocarcinoma. *J. Reticuloendothel. Soc.*, **26**, 417.

HASKILL, J. S., BECKER, S., FOWLER, W. & WALTON, L. (1982) Mononuclear cell infiltration in ovarian cancer. I. Inflammatory cell infiltrates from tumour and ascites material. *Br. J. Cancer*, **45**, 728

HAYRY, P. & TOTTERMAN, T. H. (1978) Cytological and functional analysis of inflammatory infiltrates in human malignant cells. I. Composition of the inflammatory infiltrates. *Eur. J. Immunol.*, **8**, 866.

HESS, A. B., GALL, S. A. & DAWSON, J. R. (1979) Inhibition of *in vitro* lymphocyte function by cyst and ascitic fluids from ovarion cancer patients. *Cancer Res.*, **39**, 2381.

HERBERMAN, R. B., HOLDEN, H. I., VARESIO, L. & 7 others. (1980) Immunologic reactivity of lymphoid cells in tumours. In *Contemporary Topics in Immunobiology*, Vol. 10 (Eds. Witz & Hanna) New York: Plenum Press, p. 61.

IOACHIM, H. L. (1976) The stromal reaction of tumours: An expression of immune surveillance. *J. Natl Cancer Inst.*, **57**, 465.

KALL, M. A. & KOREN, H. S. (1978) Heterogeneity of human natural killer cell populations. *Cell Immunol.*, **40**, 58.

KLEIN, E., VANKY, F., GALILI, U., VOSE, B. M. & FOPP, M. (1980) Separation and characteristics of tumour-infiltrating lymphocytes in man. In *Contemporary Topics in Immunobiology*, Vol 10 (Eds Witz & Hanna) New York: Plenum Press, p. 79.

KOREN, H. S., AMOS, D. B. & BUCKLEY, R. H. (1978) Natural Killing in immunodeficient patients. *J. Immunol.*, **120**, 796.

KOREN, H. S., ANDERSON, S. J. & ADAMS, D. O. (1981) Studies on the antibody-dependent cell mediated cytotoxicity (ADCC) of thioglycollate-stimulated and BCG-activated peritoneal macrophages. *Cell. Immunol.*, **57**, 51.

KOSTER, F. T., MCGREGOR, D. D. & MACKANESS, G. B. (1971) The mediator of cellular immunity. II. Migration of immunologically committed lymphocytes into inflammatory exudates. *J. Exp. Med.*, **133**, 409.

LOVCHIK, J. & HONG, R. (1977) Antibody-dependent cell-mediated cytolysis (ADCC): Analysis and projections. *Prog. Allergy*, **22**, 1.

MANTOVANI, A., GIUSEPPE, P., POLENTARUTTI, N., BOLIS, G., MANGIONI, C. & SPREAFICO, F. (1979) Effects on *in vitro* tumor growth of macrophages isolated from human ascites ovarian tumors. *Int. J. Cancer*, **23**, 157.

MANTOVANI, A., POLENTARUTTI, N., PERI, G. & 4 others (1980) Cytotoxic activity on tumor cells of peripheral blood monocytes and tumor-associated macrophages in patients with ascitic ovarian tumors. *J. Natl. Cancer Inst.*, **64**, 1307.

MANTOVANI, A., ALLAVENA, P., SESSA, C., BOLIS, G. & MANGIONI, C. (1980) Natural killer activity of lymphoid cells isolated from human ascitic ovarian tumors. *Int. J. Cancer*, **25**, 573.

MORETTA, K., FERRARINI, M. & COOPER, M. D. (1978) Characterization of human T-cell sub-populations as defined by specific receptors for immunologists. In *Contemporary Topics in Immunobiology*, Vol. 8 (Eds. Warner & Cooper). New York: Plenum Press, p. 19.

MULE, J. J., JONES, F. R. HELLSTRÖM, I. & HELLSTRÖM, K. E. (1979) Selective localization of radiolabelled immune lymphocytes into syngeneic tumors. *J. Immunol.*, **79**, 600.

POULTON, T., CROWTHER, M. E. & HAY, F. C. (1978) Immune complexes in ovarian cancer. *Lancet*, ii, 72.

RUSSELL, S. W., GILLESPIE, G. Y. & PACE, J. L. (1980) Evidence for mononuclear phagocytes in solid neoplasms and appraisal of their non-specific cytotoxic capabilities. In *Contemporary Topics in Immunobiology*, Vol. 10 (Eds. Witz & Hanna) New York: Plenum Press. p. 79.

SAKSELA, E., TIMONEN, T., RANKI, A. & HAYRY, P. (1979*a*) Morphological and functional characterization of isolated effector cells responsible for human natural killer activity to fetal fibroblasts and to cultured cell line targets. *Immunol. Rev.*, **44**, 71.

SAKSELA, E. T., TIMONEN, T. & CANTELL, K. (1979*b*) Human natural killer cell activity is augmented by interferon via recruitment of "pre-NK" cells. *Scand. J. Immunol.*, **10**, 257.

SNYDERMAN, R., PIKE, M. C., FISCHER, D. B. & KOREN, H. S. (1977) Biologic and biochemical activities of continuous macrophage cell lines P388D and J774.1. *J. Immunol.*, **119**, 2060.

TOTTERMAN, T. H., PARTHENAIS, E., HAYRY, P., TIMONEN, T. & SAKSELA, E. (1980) Cytological and functional analysis of inflammatory infiltrates in human malignant tumors. III. Further functional investigations using cultured autochthonous tumour cell lines and freeze-thawed infiltrating inflammatory cells. *Cell. Immunol.*, **54**, 219.

UNDERWOOD, J. C. E. (1974) Lymphoreticular infiltration in human tumours: Prognostic and biological implications: A review. *Br. J. Cancer*, **30**, 538.

VOSE, B. M., VANKY, F. ARGOV, S. & KLEIN, E. (1977). Natural cytotoxicity in man: Activity of lymph node and tumor-infiltrating lymphocytes *Eur. J. Immunol.*, **7**, 753.

VOSE, B. M. & MOORE, M. (1979) Suppressor cell activity of lymphocytes infiltrating human lung and breast tumours. *Int. J. Cancer*, **24**, 579.

WERKMEISTER, J. A., PIHL, E., NIND, A. P., FLANNERY, G. R. & NAIRN, R. C. (1979) Immunoreactivity by intrinsic lymphoid cells in colorectal carcinoma. *Br. J. Cancer*, **40**, 839.

ZARLING, J. M., SCHLAIS, J., ESKRA, L., GREEN, J. J., TS'O. P. O. P. & CARTER, W. A. (1980) Augmentation of human natural killer cell activity by polyinosinic acid-polycytidylic acid and its nontoxic mismatched analogues. *J. Immunol.*, **124**, 1852.